# Fragile science

**DOI:** 10.1128/mbio.00746-24

**Published:** 2024-04-03

**Authors:** Arturo Casadevall, Ferric C. Fang

**Affiliations:** 1 Department of Molecular Microbiology and Immunology, Johns Hopkins School of Public Health, Baltimore, Maryland, USA; 2 Departments of Laboratory Medicine, Pathology and Microbiology, University of Washington, Seattle, Washington, USA

**Keywords:** science, fragile, policy

## Abstract

Science currently faces major external and internal threats. External threats include persistent anti-science attacks, the post-pandemic politicization of public health, and chronic underfunding. Internal threats include a proliferation of low-quality studies, an epidemic of retractions, and questions regarding the reproducibility of important research findings. These threats occur just as humanity faces an unprecedented onslaught of existential challenges including climate change, a failing green revolution, pandemics, and severe environmental degradation of the planet, each of which will require scientific solutions. History shows that science is fragile and vulnerable to theocratic, ideological, and authoritarian forces. In this moment of crisis, it is important for all scientists to become foot soldiers in the defense of science.

## EDITORIAL

*When you look at a piece of delicately spun glass you think of two things: how beautiful it is and how easily it can be broken*.

—Tennessee Williams, production notes for *The Glass Menagerie*

The COVID-19 pandemic has provided incontrovertible evidence of the power of science to help humanity. When the new coronavirus SARS-CoV-2 burst forth into human populations, unleashing the worst infectious disease emergency since the 1918 influenza pandemic, initial cases were associated with high mortality. Countries quickly moved to protect their citizens by closing borders, triggering a downturn in commercial activity that soon became a worldwide economic crisis. However, in contrast to the 1918 influenza pandemic, society had a powerful defense in the form of a biomedical establishment with an advanced research capacity and a well-developed pharmaceutical industry that rose to the challenge. The genomic sequence of the virus was determined within weeks of its discovery, which rapidly led to nucleic acid amplification tests for diagnosis. Within a year, vaccines were deployed to protect the most vulnerable individuals, and COVID-19 was being successfully treated with small-molecule antivirals and antibody-based therapies in the form of convalescent plasma and monoclonal antibodies. Consequently, mortality fell rapidly, and by 2021, the world was on its way to recovery.

The response of the scientific community to the emergence of SARS-CoV-2 saved untold lives and greatly ameliorated suffering relative to what could have been. In the wake of this success, one might think that scientists would be in a celebratory mood and that societal support for science would be strong. Unfortunately, the response to the pandemic became politicized, and anti-science forces became energized (1, 2). The anti-vax movement fanned the flames of skepticism, leading to reduced rates of vaccination (3). As we write this essay, we are witnessing new outbreaks of measles in the United States, a viral disease that can kill children but can be completely prevented by a safe and effective vaccine. Some are blaming the origin of the COVID-19 pandemic on science, based on speculation that the virus originated from an accidental lab-leak or a malicious act of genetic engineering (4). Such attacks on biomedical scientists add to decades of ongoing attacks on climate scientists, with various interest groups denying that anthropogenic gas emissions are warming the planet even as temperatures continue to rise. The scientific enterprise also continues to suffer from chronic underfunding, as a result of political resistance to investment in research and development (5).

In addition to these external threats, internal threats to science are driven by problems within the scientific enterprise. In this third decade of the 21st century, a century that has been called the “biological century,” the biomedical sciences are facing a set of problems that collectively constitute a crisis. These problems include workforce imbalances, replication problems, an epidemic of retracted and fraudulent papers, and the proliferation of low-quality science. Such problems were all too apparent during the COVID pandemic, as standards for publication became lax, ineffective treatments like hydroxychloroquine or ivermectin were advocated, and more than 400 COVID-related studies were retracted or withdrawn, including some due to egregious fraud (6–8).

With the products of science all around us, and a global scientific workforce numbering in the millions, it may strike some readers as absurd to describe science as “fragile.” However, the scientific enterprise is dependent on support from the society it serves, and there are many competing sources of information, including intuition, personal experience, authority, community, and alas, social media. COVID has reinforced our appreciation that societal support for science is fragile and must not be taken for granted.

History provides many examples of the fragility of science. From the 8th through 13th centuries, the Islamic world was at the global vanguard of technology and science. However, the increasing influence of traditionalist religious leaders in these societies was paralleled by a decline in scientific activity (9). At the dawn of the scientific revolution, new views of the solar system were also opposed by religious authorities, so that Copernicus had to publish *De revolutionibus orbium coelestium* posthumously in 1543 to avoid retribution by the clergy. The following century witnessed the trial of Galileo by the Inquisition for his support of the heliocentric model of the solar system, forcing him to recant his views. More recently, Germany surrendered its position as the leading scientific nation in the 1930s through purges of Jewish scientists driven by racist ideology and an embrace of so-called “Aryan Physics” (10). Authoritarian views grounded in communist ideology led to the suppression of biological science and genetics by Lysenko in the Soviet Union and the death of rival botanist Nikolai Vavilov (11). Similarly, the Cultural Revolution devastated science research and education in China in the 1960s (12). These examples show that science is vulnerable to theocratic, ideological, authoritarian, and racist policies. Whenever the findings of science challenge the social or political order, scientists can expect to come under attack.

External and internal threats to science can synergistically act to undermine the scientific enterprise. Funding pressures create a misalignment of incentives that can lead to bad science and misconduct. Theocratic and ideological pressures can force scientists to abandon certain lines of investigation. Given that all knowledge is connected, this can impede progress in unforeseen ways. Research misconduct and low-quality or irreproducible work provide fodder for attacks from anti-science forces. External and internal threats are thus linked and must be countered together.

In the 21st century, humanity faces a number of existential threats in addition to pandemic viruses, ranging from runaway climate change (13) to rising global demand for food (14) and ever-looming concerns about asteroids and volcanism (just ask the dinosaurs) (15). Belligerent governments are once again taking the world to the brink of nuclear war (16). Given that the human population is expected to peak in mid-century, one can anticipate even greater demands for food, water, and resources on a planet that is already highly degraded by anthropogenic activities (17). Fortunately, humanity has science, which could ameliorate some of these threats. For example, science and technology can deliver clean sources of energy, remove carbon from the atmosphere, generate drought- and pest-resistant crops, and create a planetary protection system for space-based threats. Already, there are nascent technologies for carbon capture, improved crops, and even defense against approaching bolides. However, for humans to meet these threats successfully, science must work more effectively than it ever has before. Meeting these challenges will be difficult enough without also having to combat misinformation and fake news.

The job of scientists is to do science and provide society with accurate information about the natural world through careful observation and experimentation. However, sustaining a healthy relationship between science and society will also require scientists to become foot soldiers who protect the scientific enterprise from both internal and external threats. Internally, the scientific enterprise must reform itself to produce better science, which may require major reforms affecting incentives and values. Externally, scientists must engage the public, media, and politicians to spread accurate information, promote science, and counteract anti-science voices. Until now, many scientists have remained on the sidelines, preferring to concentrate on their work and hope that the problems will go away. However, with the survival of humanity on the line, all scientists must take a stand on behalf of science.

Science is not perfect, and scientists make mistakes. Scientific work always entails uncertainty, which has not always been effectively communicated to the public. Nevertheless, science remains humanity’s best insurance policy. The contract between scientists and society is fragile and must be nurtured and protected. Although society may not be in imminent danger of losing science altogether, it behooves all scientists to recognize that science is not the only way in which the public obtains information. A failure to act will risk losing the competition to less reliable sources. Science is beautiful and worth fighting for.
